# Editorial Statement

**DOI:** 10.1093/function/zqac014

**Published:** 2022-03-17

**Authors:** Ole H Petersen

**Affiliations:** School of Biosciences, Sir Martin Evans Building, Cardiff University, Wales CF10 3AX, UK

The Russian criminal war against Ukraine has been widely condemned and institutions and individuals with close links to the Russian Government have been sanctioned by the US, the EU and the UK. There have been calls for a boycott of all Russian research and even for scientific journals to refuse publishing papers from Russian institutions. Although *Function*, like all civilized organisations, condemns the brutal and unprovoked Russian invasion of Ukraine, this journal continues to work for the global exchange of scientific knowledge, following the guidelines set forth by the World Association of Medical Editors. Editorial decisions are not affected by the origins of the manuscript, including the nationality, ethnicity, political beliefs, race, or religion of the authors, among other protected characteristics, and therefore we have no plans to prohibit publication of papers that pass our stringent peer-review standards simply because they are co-authored by colleagues from Russia. In this issue of *Function*, we publish three articles co-authored by scientists working at Moscow State University (also called Lomonosov Moscow State University). At the same time, and in the strongest possible terms, we condemn the belligerent public statement issued by the Russian Union of Rectors that openly and explicitly supports the illegal war against Ukraine. We note that the Rector of Lomonosov Moscow State University is President of the Russian Union of Rectors and, as the first signatory to the statement, has actively encouraged this inexcusable war.

